# Paraganglioma of Urinary Bladder Managed by Laparoscopic Partial Cystectomy in Conjunction with Flexible Cystoscopy: A Case Report

**DOI:** 10.1089/cren.2017.0132

**Published:** 2018-02-01

**Authors:** Hossam S. El-Tholoth, Sami Al Rasheedi, Faris Alharbi, Waleed Alshammari, Tarek Alzahrani, Ahmed Al Zahrani

**Affiliations:** Department of Urology, Prince Sultan Military Medical City, Riyadh, Saudi Arabia.

**Keywords:** paraganglioma of urinary bladder, laparoscopic partial cystectomy, cystoscopy

## Abstract

***Background:*** Paraganglioma of the urinary bladder (PUB) is exceedingly rare, accounting for <0.1% of all urinary bladder tumors. Various challenging treatment options are available.

***Case Presentation:*** A 67-year-old female presented with malignant hypertension on four medications for which investigation was done. An observation of having functioning PUB was noted. She was admitted and laparoscopic partial cystectomy was done with the guidance of flexible cystoscopy. She had a smooth postoperative course and was discharged home, then catheter was removed after cystogram. Histopathology confirmed the diagnosis of a bladder paraganglioma. Finally, during the last follow-up, the patient was asymptomatic with controlled blood pressure and normalized catecholamine levels with no evidence of recurrence.

***Conclusion:*** PUB is an exceedingly rare tumor that can be managed with minimally invasive techniques such as laparoscopic partial cystectomy with cystoscopy guidance.

## Introduction

Paragangliomas of the urinary bladder (PUB) is remarkably rare, accounting for <0.1% of all urinary bladder tumors.

## Case Presentation

**FIG. 1.**
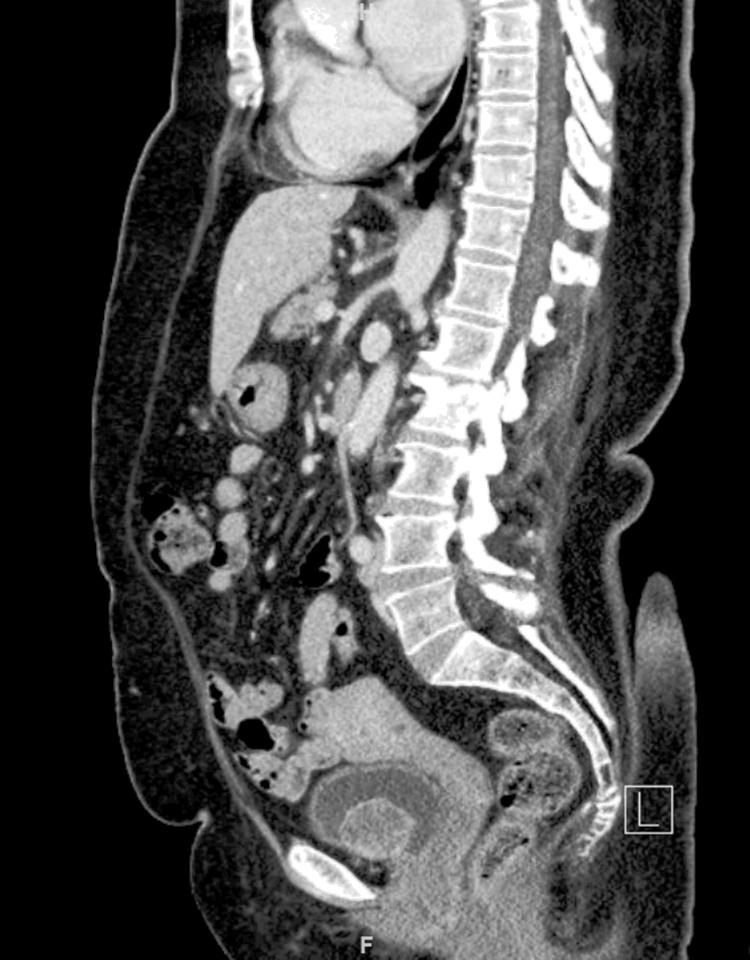
Sagittal reconstructed CT scan image showing bladder mass.

**FIG. 2.**
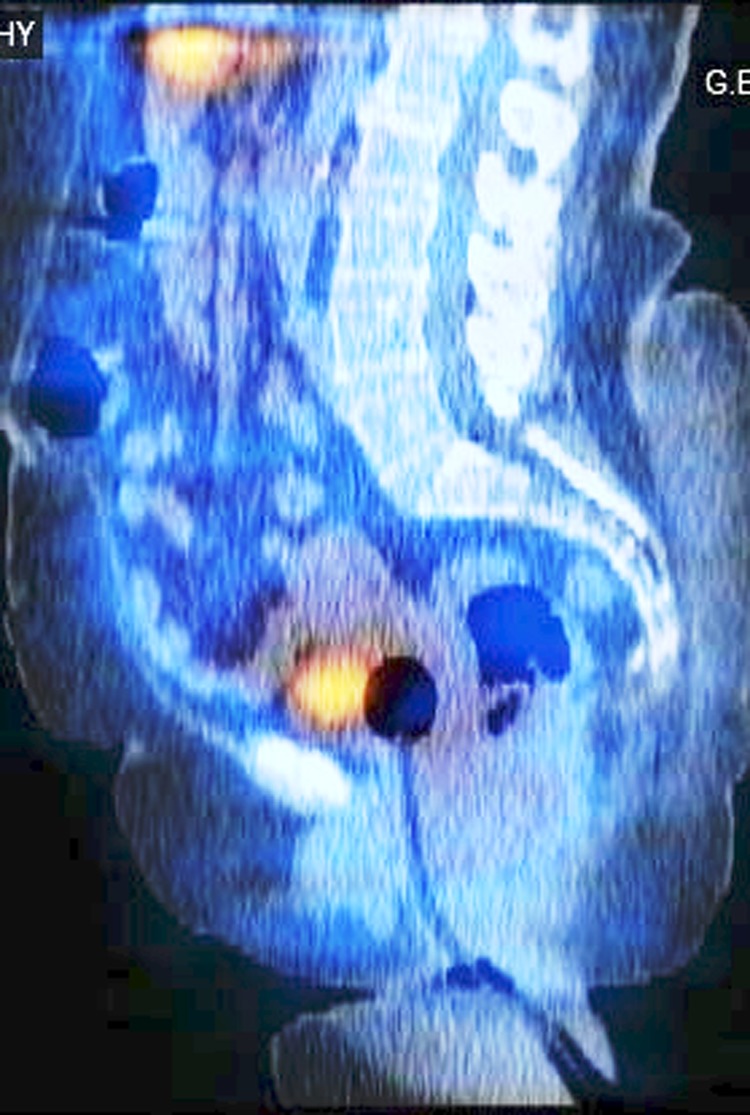
MIBG scintigraphy—sagittal view showing bladder mass. MIBG, metaiodobenzylguanidine scan.

A 67-year-old female presented with malignant hypertension on four medications for which investigation by an endocrinologist showed positive metaiodobenzylguanidine scan (MIBG), elevated catecholamines levels, and CT with contrast showed intravesical tumor with a tentative diagnosis of bladder paraganglioma of size 3.5 × 3 cm as seen in Figure 1 and Figure 2.

**FIG. 3.**
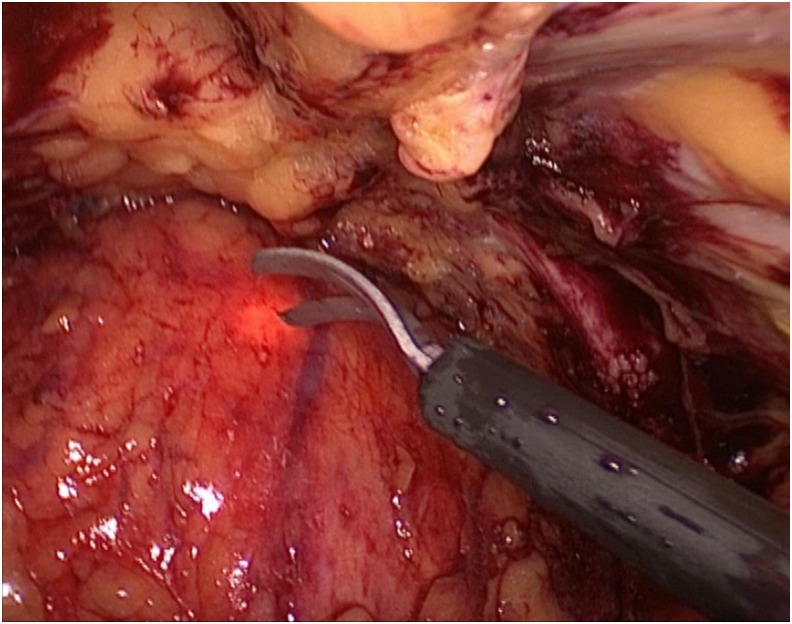
Laparoscopic view to start resection over guidance of cystoscopy light.

**FIG. 4.**
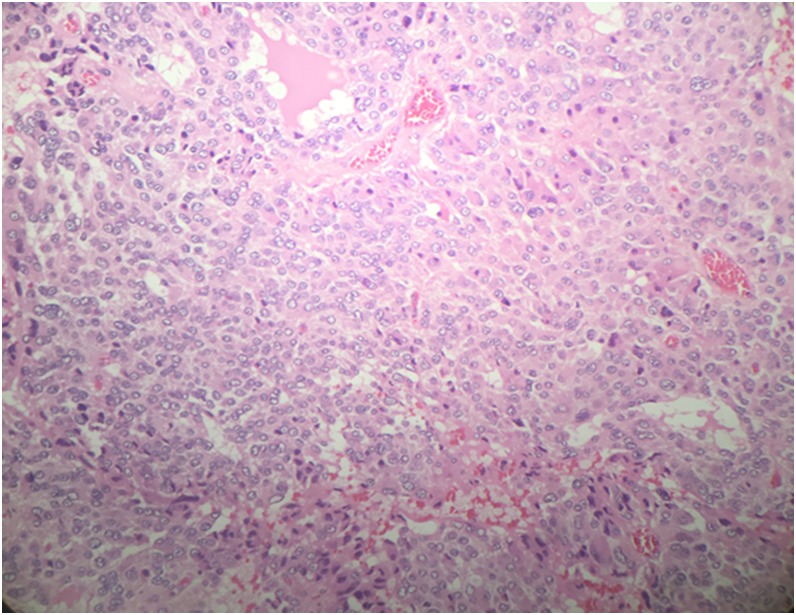
Histopathology slide of the lesion confirming the diagnosis of bladder paraganglioma.

She was admitted and was prepared for surgery. Laparoscopic partial cystectomy was conducted (she was placed in low lithotomy position and laparoscopic procedure was started by mobilizing the bladder in combination with flexible cystoscopy that showed a mucosal bulge located in anterior bladder wall with a wide base near the bladder neck. The light of the endoscope guided the start of resection with a safety margin and then we excised the mass completely with a good safety margin as shown in Figure 3, and bladder was closed in one layer laparoscopically. During surgery, she developed one attack of hypertension and was managed by anesthesia with no intraoperative or immediate postoperative complications. She had a smooth postoperative course and was discharged home, then the catheter was removed after cystogram. Histopathology confirmed the diagnosis of bladder paraganglioma (Fig. 4). As well, a follow-up at 1 year postoperation, the patient was asymptomatic with controlled blood pressure and normalized catecholamines level with no evidence of recurrence.

## Conclusion

PUB is an exceedingly rare tumor that can be managed with minimally invasive techniques such as laparoscopic partial cystectomy with cystoscopy guidance.

## Discussion

PUB is rare <1% of bladder tumors and 6% of all paragangliomas. Out of these PUB, only 10% show malignant behavior. There are no specific sites inside the urinary bladder more vulnerable to PUB. However, it is commonly reported at the lateral wall, posterior wall, and trigone. PUB can be misdiagnosed with other urothelial bladder tumors. Most of the reported cases are younger than those of urothelial carcinoma with a median age of 43 years.^1,2^ In our case study, the patient is a 67-year-old coping with the median age of other urothelial tumors. The clinical presentation of functioning paraganglinoma is manifested by a headache and uncontrolled hypertension despite medications. The use of MIBG and elevated urinary catecholamine levels help the tentative diagnosis of the case. Immunohistochemistry and characteristic histologic features are the cornerstones for confirmative diagnosis with high index of clinical suspicion.^2^ There are various treatment options for PUB, including transurethral resection, partial cystectomy, and radical cystectomy.^3^ Because the paraganglionoma of the urinary bladder in our case was preoperatively known to be functioning, a large size, and located in the anterior bladder wall, laparoscopic partial cystectomy was the preferred option of treatment, as intraoperatively uncontrolled hypertension may be disastrous in such cases. To the best of our knowledge, the only laparoscopically assisted partial cystectomy for a known preoperative functional paraganglinoma that was effectively treated without sequels under cystoscopic guidance was previously reported by Gofrit et al.^4^ for open partial cystectomy.

## Abbreviations Used

MIBG: metaiodobenzylguanidine scan
PUB: paraganglioma of urinary bladder
